# Reliability of Mental Workload Index Assessed by EEG with Different Electrode Configurations and Signal Pre-Processing Pipelines

**DOI:** 10.3390/s23031367

**Published:** 2023-01-26

**Authors:** Alfonso Mastropietro, Ileana Pirovano, Alessio Marciano, Simone Porcelli, Giovanna Rizzo

**Affiliations:** 1 Institute of Biomedical Technologies, National Research Council, Via Fratelli Cervi 93, 20054 Segrate, Italy; 2 Department of Molecular Medicine, University of Pavia, Via Forlanini 6, 27100 Pavia, Italy

**Keywords:** mental workload, EEG, signal processing, reliability, cognitive performance, Simon task

## Abstract

Background and Objective: Mental workload (MWL) is a relevant construct involved in all cognitively demanding activities, and its assessment is an important goal in many research fields. This paper aims at evaluating the reproducibility and sensitivity of MWL assessment from EEG signals considering the effects of different electrode configurations and pre-processing pipelines (PPPs). Methods: Thirteen young healthy adults were enrolled and were asked to perform 45 min of Simon’s task to elicit a cognitive demand. EEG data were collected using a 32-channel system with different electrode configurations (fronto-parietal; Fz and Pz; Cz) and analyzed using different PPPs, from the simplest bandpass filtering to the combination of filtering, Artifact Subspace Reconstruction (ASR) and Independent Component Analysis (ICA). The reproducibility of MWL indexes estimation and the sensitivity of their changes were assessed using Intraclass Correlation Coefficient and statistical analysis. Results: MWL assessed with different PPPs showed reliability ranging from good to very good in most of the electrode configurations (average consistency > 0.87 and average absolute agreement > 0.92). Larger fronto-parietal electrode configurations, albeit being more affected by the choice of PPPs, provide better sensitivity in the detection of MWL changes if compared to a single-electrode configuration (18 vs. 10 statistically significant differences detected, respectively). Conclusions: The most complex PPPs have been proven to ensure good reliability (>0.90) and sensitivity in all experimental conditions. In conclusion, we propose to use at least a two-electrode configuration (Fz and Pz) and complex PPPs including at least the ICA algorithm (even better including ASR) to mitigate artifacts and obtain reliable and sensitive MWL assessment during cognitive tasks.

## 1. Introduction

Mental workload (MWL) can be defined, as recently proposed by Longo et al. [1], as “the degree of activation of a finite pool of resources, limited in capacity, while cognitively processing a primary task over time, mediated by external stochastic environmental and situational factors, as well as affected by definite internal characteristics of a human operator, for coping with static task demands, by devoted effort and attention”. Even if the latter seems, to date, the most comprehensive definition of MWL, more commonly, MWL is roughly defined as a multidimensional construct describing the relationship between the cognitive task demand, under specific conditions, and the actual resources that can be actively engaged by an individual during the execution of the task [2,3].

MWL is a relevant construct since it is involved in almost all human activities [4], from everyday life activities to the most complex cognitive tasks, when a certain degree of mental processing is required. Interestingly, MWL is correlated to task demand and performance, since it is usually considered that high, as well as low, levels of MWL may have a negative impact on task performance and increase the incidence of errors [5,6,7] during the execution of a task. Therefore, the assessment and quantification of MWL represent one of the main interests in ergonomics [8] with relevant potential impact in different fields such as aeronautics [9], automotive [10], education and training [11], clinical practice, and rehabilitation [12,13].

Among all the available assessment methods, physiological measurements have been proven to provide an objective and minimally invasive evaluation with high reliability. These techniques estimate MWL from changes in biological signals and their derived variables that are related to the cardiovascular and respiratory system, the ocular responses, and the electrodermal and brain activity [1,14].

In this context, electroencephalography (EEG) is a widely used technique for the estimation of MWL, since it allows obtaining a direct non-invasive measurement of brain activity in different conditions. The study of changes occurring within the characteristic EEG oscillation rhythms during the execution of specific tasks has revealed that an increase in MWL is associated with a decrease in alpha activity (8–13 Hz) in the parietal brain area and an increase in theta activity (4–8 Hz) in the frontal area [15,16,17]. In particular, a correlation between increased task complexity and the power spectra of EEG signals recorded at midline electrodes has been observed [18]. For this reason, a simple metric to quantify the MWL is the theta-to-alpha ratio, which is calculated by dividing the theta band power over the EEG midline frontal channel (Fz), and the alpha band power over the parietal channel (Pz) [18,19,20]. However, EEG signals have also been used to estimate the MWL, with different configurations and number of sensors, (e.g., CZ, pre-frontal and lateral fronto-parietal electrodes) according to the experimental setup [21]. In this scenario, to the best of our knowledge, a systematic evaluation of the influence of the employed electrodes on the quantitative estimation of MWL during a cognitive task is still lacking.

Another key factor that can influence the quantification of MWL from EEG power spectra is the pre-processing pipeline applied to remove the extracerebral components that affect the EEG recording [22]. Various pre-processing methods are currently proposed in the literature to extract MWL indicators from EEG signals, and a consensus is still missing among researchers. The bandpass filtering is typically used in most papers but with different cut-off frequencies [20,23,24]; the major artifacts are typically removed with Independent Component Analysis (ICA) [25,26], Artifact Subspace Reconstruction (ASR) algorithms [20,27] or other methods [28]; the signal is mainly re-referenced to the average of the electrodes [23,27] or the average of the mastoid electrodes [20]; the channel rejections are performed automatically [29] or manually [30]. Although some more general pipelines for EEG signal analysis exist, they are quite broad and not universally adopted [31]. Furthermore, they are not always suitable for real-time applications implying MWL estimation, since they are based on quite complex methods that are often time-consuming and do not allow automatic real-time analysis.

In recent years, there has been a relevant growth in EEG analysis methods. The high amount of available new tools leads to the need of developing guidelines to pursue research reproducibility and the robustness of results to increase consistency within the scientific literature. This issue is now being referred to as the “reproducibility crisis” [32]. Indeed, a lack of consistent EEG signal pre-processing techniques can affect the comparison of quantitative results from different studies, even if the same dataset is analyzed. The reliability of EEG biomarkers is particularly critical in the perspective of employing them in clinical practice for understanding human cognition [33,34]. As underlined in the Organization for Human Brain Mapping reports [35] in presenting best practices for specific neuroimaging methods, a single best analysis workflow does not exist, and the optimal solution has to be adapted for the specific application [34]. In the specific field of MWL estimation, the literature mainly focused on test–retest reliability in longitudinal studies and on the effects of EEG signal pre-processing on the performances of automatic MWL-level classification algorithms [36]. However, to the best of our knowledge, no evaluation has been systematically conducted on the MWL quantification by EEG biomarkers, i.e., the theta-to-alpha ratio values, disregarding the automatic load classification problem.

Considering what was previously introduced, the main aim of the paper is to evaluate the reliability of MWL assessment by EEG in terms of reproducibility and sensitivity to identify the best processing pipeline and electrode configuration for MWL quantification during cognitive tasks.

## 2. Related Works

The reliability of EEG analysis, and consequently the quantitative indexes derived, is a long-standing fundamental issue addressed by the scientific community. In the literature, test–retest studies have been conducted to assess the replicability of EEG-derived indexes over time. Ding and colleagues [37] tested the reproducibility of EEG spectral analysis at the electrode and source level during rest and imaginary tasks. Corsi-Cabrera et al. [38] conducted a longitudinal study on six women to assess within-subject reliability and inter-session stability of resting EEG over nine months in the estimation of the absolute power and inter- and intra-hemispheric coherent activity. However, these works did not take into consideration the effects of different pre-processing workflows on the results’ replicability. In this context, a few works have tested the pre-processing influence on the longitudinal replicability of results. In 2017, Shirk et al. [39] tested the impact of subjective artifact removal on Event-Related Potential (ERP) results, estimating the inter-rater reliability of different subjective signal-cleaning approaches. The test–retest study by Suarez-Revelo and colleagues [40,41] compared different pre-processing of resting state EEG for the estimation of spectral power in six frequency bands. For specific MWL correlates estimation, a test–retest study was conducted in 2021 by Getzmann et al. [42] to assess the performance of the cEEGrids recordings, which are based on C-shaped electrode arrays positioned around the ear. However, no evaluation as regards the pre-processing technique was presented.

While the test–rest approach is valid to prove the stability of results, especially in longitudinal studies, it is not the most suitable test to assess the impact of pre-processing on quantitative estimation when repeated measurements are not provided. In this context, a series of papers have been recently published in which the performances of machine learning approaches to classify the MWL level after different signal pre-processing pipelines were compared [36,43,44,45,46,47,48,49,50,51]. These works are focused only on the automatic classification accuracy, considering several features extracted from all the EEG frequency bands and electrode signals, e.g., ERP, as input to the algorithm, whereas any direct evaluation of the EEG features extracted is provided.

To the best of our knowledge, in the published literature, no works are investigating how the pre-processing workflow choices affect the MWL quantitative correlates, i.e., the theta-to-alpha ratio tested in the present work.

## 3. Materials and Methods

### 3.1. Experimental Protocol

**Figure 1:**
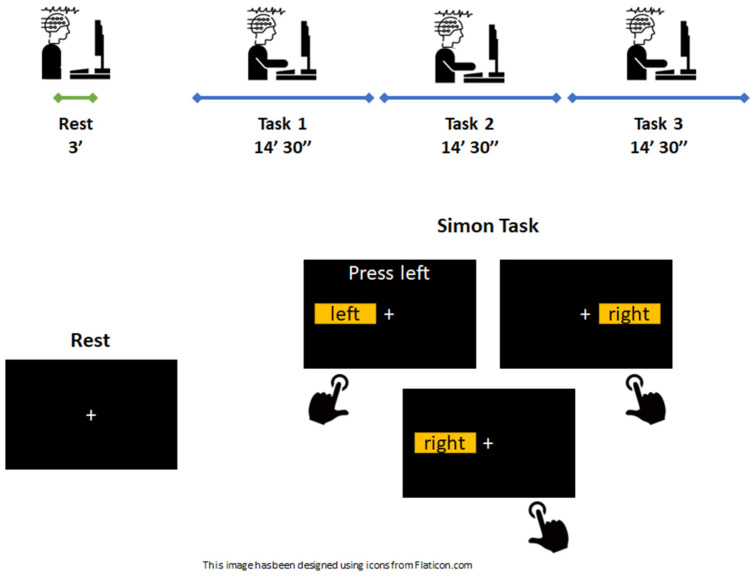
A schematic representation of the experimental protocol timing (**upper panel**) and an example depicting what was presented to the volunteers on the PC screen (**lower panel**).

Thirteen young healthy adults (age: 27 ± 6; 9 males/4 females) were enrolled in the study. The study was conducted according to the principles expressed in the Declaration of Helsinki and was approved by the local ethics committee of the University of Pavia, Italy (2531CEMaugeri-27072021). The participants signed a written informed consent. Subjects were asked to avoid ingesting any caffeine-containing drink or nicotine and performing mentally demanding tasks for at least 3 h before the session started. Moreover, they were invited to sleep at least 7 h before the experiment. The volunteers were not allowed to take any medication before the experimental session, and they did not suffer from any type of neurological and psychiatric disease. The experiments were performed at controlled room temperature (18–20 °C) and air humidity (40–60%). The experimental session consisted of performing a 45 min cognitive-demanding task sitting in front of a computer screen, and it was composed of three consecutive blocks of 14 min and 30 s each (i.e., Task 1, Task 2, Task 3), interspersed with 30 s of rest. At the beginning of the sessions, a 3-min resting period with open eyes was proposed to the volunteers and used as a baseline signal. Simon’s task was selected to elicit a cognitive demand in the volunteers. The Simon task is a behavioral measure of interference/conflict resolution [52,53]. The participants were asked to respond to visual stimuli by pressing a rightward keyboard button to the “right” stimulus and a leftward button to the “left” stimulus. The stimuli were randomly presented on the right side or the left side of the screen. Regardless of the spatial presentation of the stimuli, the subjects were asked to press the buttons corresponding to the letter shown by the visual stimulus. A schematic representation of the experimental protocol is displayed in Figure 1. Cognitive tasks were implemented and presented online using the PsyToolkit platform [54,55] (https://www.psytoolkit.org, accessed on 20 January 2023). To measure users’ performance, Reaction Times (RT) and Error Rates (ERR%) were collected as behavioral data in the different blocks of tasks.

### 3.2. EEG Acquisitions

Continuous EEG data were collected using a compact 32-channel system (eego™sports 32, ANT Neuro^®^, Enschede, The Netherlands). A gel-based electrode cap with sintered Ag/AgCl electrodes was used (Waveguard, ANT Neuro^®^, 10–20 system). The online reference was placed at the CPz electrode. Signal was acquired with eego sports acquisition software connected to a 24 bits amplifier at a sampling rate of 500 Hz. Impedances for all electrodes were kept below 20 kΩ. EEG signals were recorded across 30 channels: Fp1, Fpz, Fp2, F7, F3, Fz, F4, F8, FC5, FC1, FC2, FC6, T7, C3, Cz, C4, T8, CP5, CP1, CP2, CP6, P7, P3, Pz, P4, P8, POz, O1, Oz, and O2 excluding the mastoids electrodes (M1 and M2). The starting and ending points of each block composing the acquisition (Rest, Task 1, Task 2, Task 3) were manually labeled using the acquisition software.

### 3.3. EEG Pre-Processing

**Figure 2:**
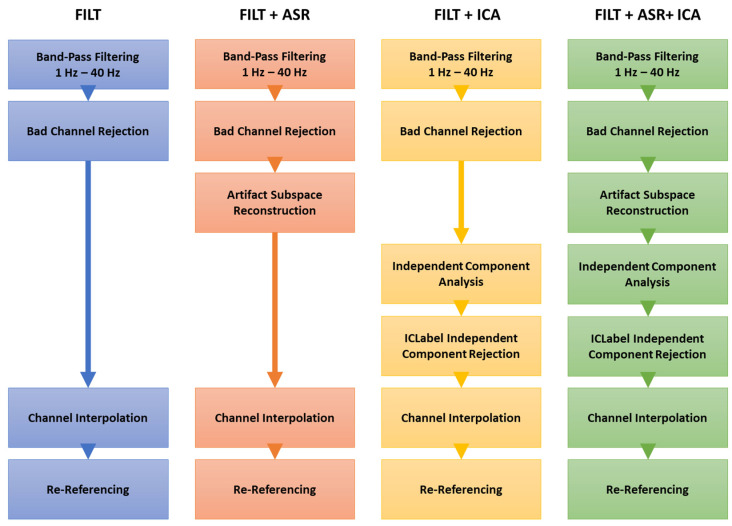
A schematic representation of the pre-processing pipelines that were evaluated in this study.

Four different processing pipelines were evaluated to assess their impact on the estimation of the MWL indicator. A schematic representation is displayed in Figure 2.

1. FILT—The first and simplest pipeline was characterized using band-pass filtering to mitigate the effects of the artifacts. In detail, EEG signals were band-pass filtered in the range 1–40 Hz using a Hamming windowed sinc FIR filter. Bad channels were removed by evaluating the normed joint probability of the average log power across the channels [56]. Channels whose probability falls more than three standard deviations from the mean are removed as bad channels.
2. FILT + ASR—The second pipeline was implemented by adding the ASR algorithm to the FILT pipeline. ASR uses principal-component-like subspace decomposition to remove transient and high-amplitude artifacts, it provides a noiseless signal reconstruction using a reference signal fragment [57] and can be helpful for real-time artifact removal. ASR was used to interpolate artifact “bursts” with a variance higher than fifteen standard deviations different from the automatedly detected reference signal, as previously suggested [58].
3. FILT + ICA—The third pipeline was proposed by adding the ICA artifact rejection method to the first pipeline. ICA algorithms are typically used to detect and remove artifacts (such as eye movements and electrocardiographic signals) that usually overlay with brain activity in EEG recordings. The extended Infomax [59] ICA algorithm was used in this work. ICLabel [60] was used to automatically reject independent components having a probability to be plausible brain sources of less than 40%.
4. FILT + ASR + ICA—The last most complex pipeline included sequentially all the previous different approaches.

To complete all the previous pipelines, channels that were removed as “bad channels” were replaced by data interpolated from nearby “artifact-free” channels using a spherical function, and EEG signals were re-referenced to the average of the channels. Among all the analyzed EEG signals, on the whole, 3 channels were removed (specifically P7 in 1 subject and CP2 in 2 subjects).

All the pre-processing steps were implemented in MATLAB (R2021b, The MathWorks) using the EEGLAB toolbox [61].

### 3.4. MWL Assessment

The pre-processed EEG signals were analyzed in the frequency domain to extract the power spectra in the range 1–45 Hz using the Welch’s power spectral density (PSD) estimate. The EEG signal was windowed using a Hamming window (1 s length, 500 samples, non-overlapping) and the periodogram was computed, for each segment, by using the discrete Fourier transform. The squared magnitude of the result was computed and the individual periodograms were averaged, separately for each of the three experimental blocks, to obtain the power spectra for each task. Subsequently, the integral of the power spectrum across frequencies in theta (4–8 Hz) and alpha (8–13 Hz) ranges was calculated to obtain the absolute band power for each channel. The MWL index of each block was then calculated by dividing the theta absolute power θ with the alpha absolute power α into three different electrode configurations.

1. Fz and Pz electrodes:
  (1) $$MWL_{Fz,Pz}=\frac{\theta_{Fz}}{\alpha_{Pz}}$$
2. Cz electrode:
  (2) $$MWL_{Cz}=\frac{\theta_{Cz}}{\alpha_{Cz}}$$
3. Frontal (F7, F3, Fz, F4, F8) and Parietal (P7, P3, Pz, P4, P8) electrodes:
  (3) $$MWL_{FP}=\frac{\theta_{Frontal}}{\alpha_{Parietal}}$$

where *θ_Frontal* and *α_Parietal* are the sum of the absolute powers in frontal and parietal electrodes.

The MWL index calculated during tasks was normalized to the value of the rest condition as follows:

(4) $$MWL=\frac{\left(MWL_{Task}-MWL_{Rest}\right)}{MWL_{Rest}}$$

### 3.5. Reproducibility Assessment

The reproducibility refers to the level of consistency and agreement in the estimation of MWL at different EEG electrode configurations and pre-processing pipelines with increasing levels of complexity. To assess the reproducibility, Intraclass Correlation Coefficient (ICC) was adopted as a descriptive statistical method. ICC reflects both the degree of correlation and the agreement between measurements [62]. The two-way mixed-effects model was selected to assess both consistency and agreement among different scenarios.

In particular, the two-way mixed effects, consistency, and single measurement ICC (3,1) index was defined as follows:

(5) $$\frac{MS_{R}-MS_{E}}{MS_{R}+\left(k-1\right)MS_{E}}$$

whereas the two-way mixed effects, absolute agreement, and single measurement ICC (2,1) index was defined as follows:

(6) $$\frac{MS_{R}-MS_{E}}{MS_{R}+\left(k-1\right)MS_{E}+\frac{k}{n}\left(MS_{C}-MS_{E}\right)}$$

where *MSR* = mean square for rows; *MSE* = mean square for error; *MSC* = mean square for columns; *n* = number of targets; *k* = number of ratings.

### 3.6. Statistical Analysis

To evaluate if there is a statistically significant interaction effect between the three within-subjects factors (pre-processing pipelines, electrode configurations, task blocks) in explaining differences in MWL metrics estimated in different conditions (e.g., electrode configurations, processing pipelines, task blocks), the repeated measure ANOVA test was adopted. Greenhouse–Geisser correction was applied to only within-subjects factors violating the sphericity assumption (with significant Mauchly’s test *p*-value, *p* ≤ 0.05).

To evaluate the sensitivity, which refers to the ability to discriminate changes in the MWL index at increasing cognitive loads and different experimental settings, multiple pairwise comparisons between groups were performed using the pairwise *t*-test, and the false discovery rate adjustment was applied to correct *p*-values. *p*-values ≤ 0.05 were considered significant. The statistical tests were performed in R (ver. 4.2.1) [63] embedded in RStudio (2022.07.1, Build 554).

## 4. Results

### 4.1. Reproducibility

Considering each specific electrode configuration individually, the consistency among MWL metrics, obtained using different pre-processing pipelines, exhibits values (averaged over tasks) higher than 0.81 in all the conditions. In particular, the mean consistency is 0.94 for FzPz, 0.94 for Cz and 0.88 for fronto-parietal configurations, respectively. The highest consistency can be observed between Filt + ASR, Filt + ICA and Filt + ASR + ICA (maximum consistency at 0.99), whereas the lowest values are those corresponding to the comparison of Filt with the other pre-processing pipelines (minimum consistency at 0.81). As to the consistency among electrode configurations, its mean values are 0.83 in the case of FzPz vs. Cz, 0.85 in the case of FzPz vs. fronto-parietal and 0.74 in the case of Cz vs. fronto-parietal configurations, respectively.

**Figure 3:**
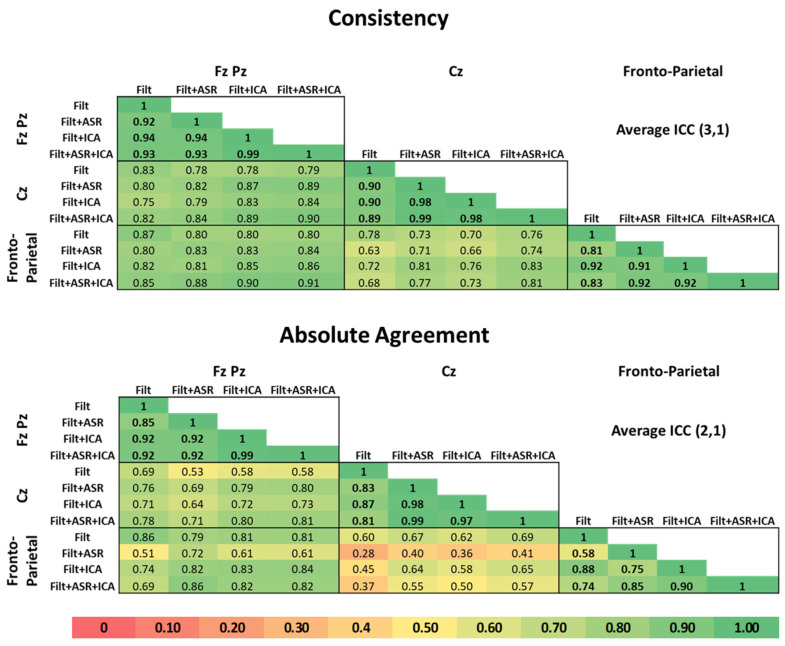
ICC values are shown for both consistency (**upper panel**) and absolute agreement (**lower panel**). Colors range from red (no consistency/absolute agreement) to green (highest consistency/absolute agreement) as shown in the color bar at the bottom of the figure.

Regarding the absolute agreement, the tendency is similar to that described above for consistency but with lower values. In particular, as regards the absolute agreement among pre-processing pipelines in each electrode’s configuration, the mean absolute agreement is 0.92 for FzPz, 0.91 for Cz and 0.78 for fronto-parietal configurations, respectively. The highest absolute agreement can be observed between Filt + ASR, Filt + ICA and Filt + ASR + ICA (maximum absolute agreement at 0.99), whereas the lowest values are those corresponding to the comparison of Filt with the other pre-processing pipelines (minimum consistency at 0.58). As to the absolute agreement among electrode configurations, its mean values are 0.73 in the case of FzPz vs. Cz, 0.77 in the case of FzPz vs. fronto-parietal and 0.49 in the case of Cz vs. fronto-parietal configurations, respectively. A concise representation of the results is shown in Figure 3.

### 4.2. Impact of Experimental Factors on MWL

To investigate the impact of the within-subjects’ factors (i.e., electrode configurations, pre-processing pipelines and tasks) in discriminating differences among MWL indexes, we explored the results of the three-way repeated measures ANOVA test (as summarized in Table 1). Considering the single factors individually (i.e., pipeline, configuration, task), significant differences within them were observed (*p* < 0.05). As to the interaction of two factors (i.e., pipeline and configuration, pipeline and task, configuration and task), statistically significant differences were shown when pipelines and configurations along with tasks, respectively, (*p* < 0.05) were considered, whereas a significant difference was not observed when the combined effect of pipelines and configuration was considered. Moreover, as shown in Table 1, there is a statistically significant three-way interaction between pipelines, configurations and tasks, F (18, 216) = 2.225, *p* = 0.004.

### 4.3. Sensitivity to MWL Changes during Prolonged Simon Task

**Figure 4:**
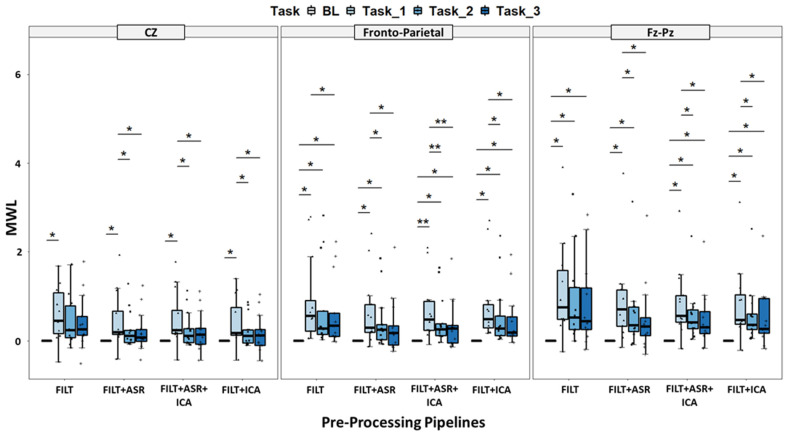
Representation of MWL in different experimental conditions (electrode configurations and pre-processing pipelines) and tasks. Asterisks refer to statistically significant differences (*p* < 0.05 *; *p* < 0.01 **).

In Figure 4, the population’s average MWL indexes calculated during the three consecutive experimental blocks, considering the three different electrode configurations and the four pre-processing pipelines, are represented. Regardless of the method/electrodes evaluated, we observe a common trend of the MWL index during the execution of the Simon task over time. Specifically, in all cases, we found an initial relevant increase in MWL compared to the rest condition in the first 15-min block of task execution. Afterward, in the second and third blocks, a decrease in MWL is observed even though it still remained higher than MWL calculated at baseline. Considering the users’ performances during the Simon task, the average RT decreases over time and blocks, ranging from 541 ± 33 ms to 515 ± 36 ms whereas, conversely, the ERR%s increases, ranging from 3.1% ± 2.1% to 4.0% ± 2.4% as shown in Figure S1.

In detail, considering the multiple pairwise comparisons results shown in Figure 4, statistically significant differences were observed in most of the conditions. In particular, exploring the differences among tasks and rest, significant MWL differences between Task 1 and Rest were found in all the electrode configurations and pre-processing approaches, whereas significant MWL differences between Task 2 and Rest were observed just in fronto-parietal and FzPz configurations considering all pre-processing pipelines. Finally, significant MWL differences between Task 3 and Rest were found in fronto-parietal and FzPz configurations in the case of FILT, FILT + ICA and FILT + ASR + ICA pipelines.

As to the differences among tasks, significant differences between Task 1 and Task 2, as well as between Task 1 and Task 3, were observed in all configurations in the case of FILT + ASR, FILT + ICA and FILT + ASR pipelines. No significant differences were found between Task 2 and Task 3.

Globally, the conditions in which the maximum number of differences (five out of six) were found are those where the fronto-parietal and FzPz electrodes are considered and the FILT + ICA and FILT + ASR + ICA pipelines were used to process the EEG signals.

A summary of descriptive statistical features and the list of *p*-values and effect sizes related to the between-groups pairwise comparisons are reported in Table S1 and Table S2, respectively.

## 5. Discussion

This paper evaluated the reproducibility of MWL estimation from EEG signals considering different processing pipelines and electrode configurations as well as the sensitivity of the MWL metric to discriminate among different cognitive loads during a prolonged cognitive task. Furthermore, this work aimed also at providing guidelines for the quantitative estimation of the MWL changes taking into consideration a few aspects that are usually overlooked in the literature and, when results are available, they lack consistency.

To assess the reliability of EEG-based MWL estimation, we requested the volunteers to perform a cognitive task, i.e., the Simon task, eliciting MWL changes related to mental processes, such as working memory and attentional control, associated with the execution of the task goal during the congruent/incongruent stimuli presentation [64]. Even though this work neglected the investigation of the neurophysiological mechanisms underlying task-related mental constructs, our results show that the Simon task was able to elicit an increase in MWL if compared to the rest condition. Furthermore, a temporal effect influences the response; in fact, the initial increase in MWL, during the first block of tasks, is followed by a reduction in the following tasks, which is probably due to the onset of mental fatigue related to the prolonged mental demand. Therefore, the MWL index appears to be sensitive to the Simon effect and its elicited changes in mental effort.

Although MWL variations were well observed in most conditions, we found a dependence of the quantification and statistical identification of changes on both acquisitions, i.e., electrode position, and pre-processing approaches. In the literature, the investigation of different electrode configurations and pre-processing pipelines focuses on the influence of these factors on MWL classification accuracy through automatic algorithms based on machine learning and deep learning [50,51]. To our knowledge, no works assessed the reliability directly in MWL indexes derived from EEG signals. This paper wants to put an accent on this quantitative aspect and provide suggestions to choose the methodological aspects that will guarantee the most reliable outcome.

Considering each single electrode configuration independently, the reproducibility expressed in terms of consistency was good or very good across all the processing pipelines used to pre-process the EEG signals in every condition. As to the absolute agreement, it exhibited lower values and moderate to very good reliability, especially in the fronto-parietal configuration. This is most likely due to the wider extension of the fronto-parietal configuration being that more prone to be corrupted by artifacts if compared to the electrodes that are placed in the midline [65]. For that reason, the MWL estimation in the fronto-parietal configuration is more susceptible to the choice of the pre-processing pipeline whereas the FzPz and Cz configurations, which exhibited the best consistency and absolute agreement among pre-processing pipelines, are less susceptible to that factor. As to the pre-processing pipelines, the most complex algorithms (e.g., FILT + ASR + ICA, FILT-ICA, and FILT-ASR) were those showing the highest values of reproducibility.

Considering the reproducibility evaluated across different electrode configurations, the lowest values of consistency and absolute agreement were found when comparing Cz with fronto-parietal configurations. Conversely, the best reliability was obtained between FzPz and fronto-parietal configuration. In general, the single electrode configuration (Cz) is that with the lowest reliability when compared to the others. Finally, even in this case, the most complex algorithms are those showing the highest consistency and agreement.

Regarding the factors that can affect the assessment of the MWL index, pre-processing pipelines and electrode configurations can be chosen independently of each other, since there is no statistically significant interaction between them. On the contrary, there is a significant interaction between tasks and electrode configurations or between tasks and pre-processing pipelines; indeed, the choice of electrode configurations and pre-processing pipelines independently affects the sensitivity of MWL to discriminate different cognitive loads during tasks.

In particular, the best electrode configurations in terms of sensitivity to MWL changes are those with the highest number of electrodes (e.g., fronto-parietal and FzPz), probing both frontal and parietal lobes. The use of Cz, even though proposed in recent work for its ease of use [20] and its potential application with single-electrode systems in real-time MWL monitoring, is not the best choice in terms of sensitivity and has the lowest reliability if compared to the other electrode configurations.

The MWL index appears to be more reliable if information is taken from both frontal and parietal electrodes rather than from a single channel probing. Indeed, the results shown in this paper support the use of at least Fz and Pz electrodes, as previously performed in other works investigating changes in MWL [18,19,66], as the minimum set of sensors suitable for obtaining reliable and sensitive estimation.

As for the pre-processing pipelines, the approaches allowing the best discrimination among tasks are those including the ICA method (with or without ASR). Our results agree with those obtained by Kingphai and Moshfeghi [50], who evaluated the accuracy of MWL classification after different signal pre-processing procedures. In fact, they found that the most complete pipelines including the ICA technique provide the best classification accuracy. However, they did not evaluate the introduction of ASR as a prior step, despite being used in other classification works [27].

A limitation to the generalization of our results could be represented by the fact that we analyzed signals obtained in a controlled experimental protocol, where subjects were requested to avoid relevant movements while performing the cognitive tasks. The influence of the pre-processing pipelines could be more significant in free-moving conditions, and the results could slightly differ from those presented in this paper. However, we assume that the midline electrode signals could provide repeatable results even in the more complex experimental setup, since movement artifacts usually less affect these electrodes. Another limitation of the present work can be represented by the low number of subjects involved but, considering that the statistical analysis pointed out significant differences even applying the correction for multiple comparisons, we are confident that the results presented in this paper could be generalized.

As for the analysis pipeline, we propose here a set of four different approaches that try to include all the pre-processing steps that are most frequently employed in the EEG literature. Anyway, variations in the choice of filters and algorithms parameters could induce different outcomes.

In the future, the evaluation of MWL reliability should be assessed also during physical exercises or free-moving experiments.

## 6. Conclusions

This work showed how the assessment of MWL using EEG signals depends on both the pre-processing pipelines and the electrode configurations. Therefore, each experimental protocol definition must be well pondered, since it can affect both the reproducibility and the sensitivity. Furthermore, comparisons of quantitative results between works implementing different methods should be carefully dealt with.

This paper suggests that using both frontal and parietal electrodes provides more robust performances in the detection of MWL changes during a cognitive task if compared to a single-electrode configuration. However, larger electrode configurations could be more prone to artifacts, be time-consuming, and be challenging in some experimental conditions (those involving non-collaborative subjects or those which involve the execution of tasks during movement).

Most complex pre-processing pipelines have been proven to be more suitable to ensure good inter-rater reliability and sensitivity in all experimental conditions.

In conclusion, our work provides a practical analysis framework for quantitative EEG-based MWL evaluation studies. We propose to use at least a two-electrode configuration (Fz and Pz) and complex pre-processing pipelines including at least the ICA algorithm (even better if ASR is included) to mitigate artifacts and obtain reliable and sensitive MWL assessment during cognitive tasks.

## Figures and Tables

**Table 1:** Summary of results of the ANOVA three-way repeated measures test. Under the “*Effect*” column are listed all the factors included in the study; *DFn* is the acronym of “degrees of freedom in the numerator”; *DFd* is the acronym of “degrees of freedom in the denominator”; F is the test statistic for ANOVA; *p* is the *p*-value; under the “***p* < 0.05**” column, there is an asterisk when the *p*-value is less than 0.05; *ges* is the “generalized eta squared”.

Effect	DFn	DFd	F	*p*	*p* < 0.05	Ges
Pipelines	1.27	15.27	4.253	0.049	*	0.016
Configurations	2	24	3.91	0.034	*	0.038
Tasks	1.49	17.9	10.47	0.002	*	0.166
Pipelines × Configurations	2.59	31.12	2.635	0.075	n.s.	0.003
Pipelines × Tasks	1.51	18.12	3.876	0.05	*	0.006
Configurations × Tasks	2.24	26.9	3.485	0.04	*	0.014
Pipelines × Configurations × Tasks	18	216	2.225	0.004	*	0.000987

## Data Availability

Data are available upon reasonable request to the corresponding author.

## Supplementary Materials

The following supporting information can be downloaded at: https://www.mdpi.com/article/10.3390/s23031367/s1.
