# Supplementary material for: Reliability of Mental Workload Index Assessed by EEG with Different Electrode Configurations and Signal Pre-Processing Pipelines
Source: Sensors (Basel). 2023 Jan 26;23(3):1367. doi: 10.3390/s23031367 (PMC9920504; doi:10.3390/s23031367)
Supplement: Supplementary file 1 [file sensors-23-01367-s001.zip › sensors-2144319-supplementary.pdf]

Table S1. Summary of the descriptive statistical features of the MWL index assessed for each group (n=13) at different pre-processing pipelines, electrode configurations and task blocks. Mad refers to median absolute deviation; SD refers to standard deviation.

Pipeline	Configuration	Task	Median	Mad	Mean	SD
FILT	Cz	Task_1	0.443	0.549	0.585	0.599
FILT	Cz	Task_2	0.237	0.304	0.409	0.537
FILT	Cz	Task_3	0.255	0.386	0.409	0.619
FILT	Frontoparietal	Task_1	0.553	0.509	0.906	0.948
FILT	Frontoparietal	Task_2	0.268	0.215	0.728	0.967
FILT	Frontoparietal	Task_3	0.335	0.356	0.613	0.783
FILT	FzPz	Task_1	0.747	0.599	1.107	1.074
FILT	FzPz	Task_2	0.521	0.436	0.882	0.964
FILT	FzPz	Task_3	0.44	0.687	0.837	0.953
FILT+ASR	Cz	Task_1	0.192	0.158	0.481	0.618
FILT+ASR	Cz	Task_2	0.105	0.214	0.253	0.41
FILT+ASR	Cz	Task_3	0.073	0.273	0.211	0.458
FILT+ASR	Frontoparietal	Task_1	0.291	0.407	0.645	0.769
FILT+ASR	Frontoparietal	Task_2	0.241	0.323	0.423	0.61
FILT+ASR	Frontoparietal	Task_3	0.166	0.392	0.329	0.639
FILT+ASR	FzPz	Task_1	0.702	0.648	0.876	0.977
FILT+ASR	FzPz	Task_2	0.348	0.478	0.611	0.821
FILT+ASR	FzPz	Task_3	0.32	0.301	0.518	0.823
FILT+ASR+ICA	Cz	Task_1	0.232	0.207	0.493	0.61
FILT+ASR+ICA	Cz	Task_2	0.104	0.232	0.26	0.391
FILT+ASR+ICA	Cz	Task_3	0.13	0.307	0.213	0.443
FILT+ASR+ICA	Frontoparietal	Task_1	0.469	0.377	0.665	0.672
FILT+ASR+ICA	Frontoparietal	Task_2	0.257	0.216	0.453	0.558
FILT+ASR+ICA	Frontoparietal	Task_3	0.268	0.468	0.365	0.563
FILT+ASR+ICA	FzPz	Task_1	0.559	0.464	0.851	0.775
FILT+ASR+ICA	FzPz	Task_2	0.411	0.353	0.579	0.591
FILT+ASR+ICA	FzPz	Task_3	0.303	0.333	0.492	0.636
FILT+ICA	Cz	Task_1	0.168	0.132	0.427	0.527
FILT+ICA	Cz	Task_2	0.103	0.228	0.217	0.348
FILT+ICA	Cz	Task_3	0.118	0.325	0.175	0.425
FILT+ICA	Frontoparietal	Task_1	0.48	0.421	0.797	0.841
FILT+ICA	Frontoparietal	Task_2	0.272	0.265	0.569	0.732
FILT+ICA	Frontoparietal	Task_3	0.178	0.292	0.466	0.611
FILT+ICA	FzPz	Task_1	0.467	0.655	0.825	0.829
FILT+ICA	FzPz	Task_2	0.357	0.301	0.582	0.648
FILT+ICA	FzPz	Task_3	0.259	0.276	0.515	0.674

Table S2. Summary of between groups pairwise t-test comparisons of MWL indexes at different pre-processing pipelines and electrode configurations. P-values (p), p-values adjusted for multiple comparisons (p adjusted) and effect size are listed.

Pipeline	Configuration	Groups Comparison			p	p adjusted	Effect size
FILT	Cz	BL	vs	Task_1	0.004	0.025*	-5.85E-01
FILT	Cz	BL	vs	Task_2	0.018	0.053	-4.09E-01
FILT	Cz	BL	vs	Task_3	0.035	0.07	-4.09E-01
FILT	Cz	Task_1	vs	Task_2	0.087	0.131	1.76E-01
FILT	Cz	Task_1	vs	Task_3	0.135	0.162	1.76E-01
FILT	Cz	Task_2	vs	Task_3	0.989	0.989	7.57E-04
FILT+ASR	Cz	BL	vs	Task_1	0.016	0.044*	-4.81E-01
FILT+ASR	Cz	BL	vs	Task_2	0.046	0.069	-2.53E-01
FILT+ASR	Cz	BL	vs	Task_3	0.122	0.146	-2.11E-01
FILT+ASR	Cz	Task_1	vs	Task_2	0.022	0.044*	2.28E-01
FILT+ASR	Cz	Task_1	vs	Task_3	0.01	0.044*	2.70E-01
FILT+ASR	Cz	Task_2	vs	Task_3	0.321	0.321	4.20E-02
FILT+ASR+ICA	Cz	BL	vs	Task_1	0.013	0.039*	-4.93E-01
FILT+ASR+ICA	Cz	BL	vs	Task_2	0.034	0.051	-2.60E-01
FILT+ASR+ICA	Cz	BL	vs	Task_3	0.108	0.13	-2.13E-01
FILT+ASR+ICA	Cz	Task_1	vs	Task_2	0.021	0.043*	2.34E-01
FILT+ASR+ICA	Cz	Task_1	vs	Task_3	0.009	0.039*	2.80E-01
FILT+ASR+ICA	Cz	Task_2	vs	Task_3	0.277	0.277	4.66E-02
FILT+ICA	Cz	BL	vs	Task_1	0.013	0.038*	-4.27E-01
FILT+ICA	Cz	BL	vs	Task_2	0.044	0.067	-2.17E-01
FILT+ICA	Cz	BL	vs	Task_3	0.162	0.194	-1.75E-01
FILT+ICA	Cz	Task_1	vs	Task_2	0.02	0.039*	2.11E-01
FILT+ICA	Cz	Task_1	vs	Task_3	0.01	0.038*	2.52E-01
FILT+ICA	Cz	Task_2	vs	Task_3	0.352	0.352	4.14E-02
FILT	Frontoparietal	BL	vs	Task_1	0.005	0.029*	-9.06E-01
FILT	Frontoparietal	BL	vs	Task_2	0.019	0.032*	-7.28E-01
FILT	Frontoparietal	BL	vs	Task_3	0.015	0.032*	-6.13E-01
FILT	Frontoparietal	Task_1	vs	Task_2	0.052	0.063	1.78E-01
FILT	Frontoparietal	Task_1	vs	Task_3	0.022	0.032*	2.93E-01
FILT	Frontoparietal	Task_2	vs	Task_3	0.229	0.229	1.15E-01
FILT+ASR	Frontoparietal	BL	vs	Task_1	0.011	0.021*	-6.45E-01
FILT+ASR	Frontoparietal	BL	vs	Task_2	0.028	0.042*	-4.23E-01
FILT+ASR	Frontoparietal	BL	vs	Task_3	0.088	0.105	-3.29E-01
FILT+ASR	Frontoparietal	Task_1	vs	Task_2	0.01	0.021*	2.21E-01
FILT+ASR	Frontoparietal	Task_1	vs	Task_3	0.004	0.021*	3.15E-01
FILT+ASR	Frontoparietal	Task_2	vs	Task_3	0.117	0.117	9.40E-02
FILT+ASR+ICA	Frontoparietal	BL	vs	Task_1	0.004	0.01**	-6.65E-01
FILT+ASR+ICA	Frontoparietal	BL	vs	Task_2	0.013	0.019*	-4.53E-01
FILT+ASR+ICA	Frontoparietal	BL	vs	Task_3	0.038	0.045*	-3.65E-01

FILT+ASR+ICA	Frontoparietal	Task_1	vs	Task_2	0.005	0.01**	2.12E-01
FILT+ASR+ICA	Frontoparietal	Task_1	vs	Task_3	0.004	0.01**	3.00E-01
FILT+ASR+ICA	Frontoparietal	Task_2	vs	Task_3	0.137	0.137	8.77E-02
FILT+ICA	Frontoparietal	BL	vs	Task_1	0.005	0.01*	-7.97E-01
FILT+ICA	Frontoparietal	BL	vs	Task_2	0.016	0.021*	-5.69E-01
FILT+ICA	Frontoparietal	BL	vs	Task_3	0.018	0.021*	-4.66E-01
FILT+ICA	Frontoparietal	Task_1	vs	Task_2	0.004	0.01*	2.28E-01
FILT+ICA	Frontoparietal	Task_1	vs	Task_3	0.004	0.01*	3.31E-01
FILT+ICA	Frontoparietal	Task_2	vs	Task_3	0.147	0.147	1.03E-01
FILT	FzPz	BL	vs	Task_1	0.003	0.016*	-1.11E+00
FILT	FzPz	BL	vs	Task_2	0.006	0.016*	-8.82E-01
FILT	FzPz	BL	vs	Task_3	0.008	0.016*	-8.37E-01
FILT	FzPz	Task_1	vs	Task_2	0.052	0.078	2.24E-01
FILT	FzPz	Task_1	vs	Task_3	0.081	0.097	2.70E-01
FILT	FzPz	Task_2	vs	Task_3	0.56	0.56	4.56E-02
FILT+ASR	FzPz	BL	vs	Task_1	0.007	0.03*	-8.76E-01
FILT+ASR	FzPz	BL	vs	Task_2	0.02	0.03*	-6.11E-01
FILT+ASR	FzPz	BL	vs	Task_3	0.043	0.051	-5.18E-01
FILT+ASR	FzPz	Task_1	vs	Task_2	0.017	0.03*	2.65E-01
FILT+ASR	FzPz	Task_1	vs	Task_3	0.013	0.03*	3.58E-01
FILT+ASR	FzPz	Task_2	vs	Task_3	0.195	0.195	9.33E-02
FILT+ASR+ICA	FzPz	BL	vs	Task_1	0.002	0.011*	-8.51E-01
FILT+ASR+ICA	FzPz	BL	vs	Task_2	0.004	0.012*	-5.79E-01
FILT+ASR+ICA	FzPz	BL	vs	Task_3	0.016	0.021*	-4.92E-01
FILT+ASR+ICA	FzPz	Task_1	vs	Task_2	0.017	0.021*	2.72E-01
FILT+ASR+ICA	FzPz	Task_1	vs	Task_3	0.012	0.021*	3.59E-01
FILT+ASR+ICA	FzPz	Task_2	vs	Task_3	0.139	0.139	8.67E-02
FILT+ICA	FzPz	BL	vs	Task_1	0.004	0.021*	-8.25E-01
FILT+ICA	FzPz	BL	vs	Task_2	0.007	0.021*	-5.82E-01
FILT+ICA	FzPz	BL	vs	Task_3	0.017	0.035*	-5.15E-01
FILT+ICA	FzPz	Task_1	vs	Task_2	0.029	0.035*	2.42E-01
FILT+ICA	FzPz	Task_1	vs	Task_3	0.023	0.035*	3.09E-01
FILT+ICA	FzPz	Task_2	vs	Task_3	0.231	0.231	6.70E-02

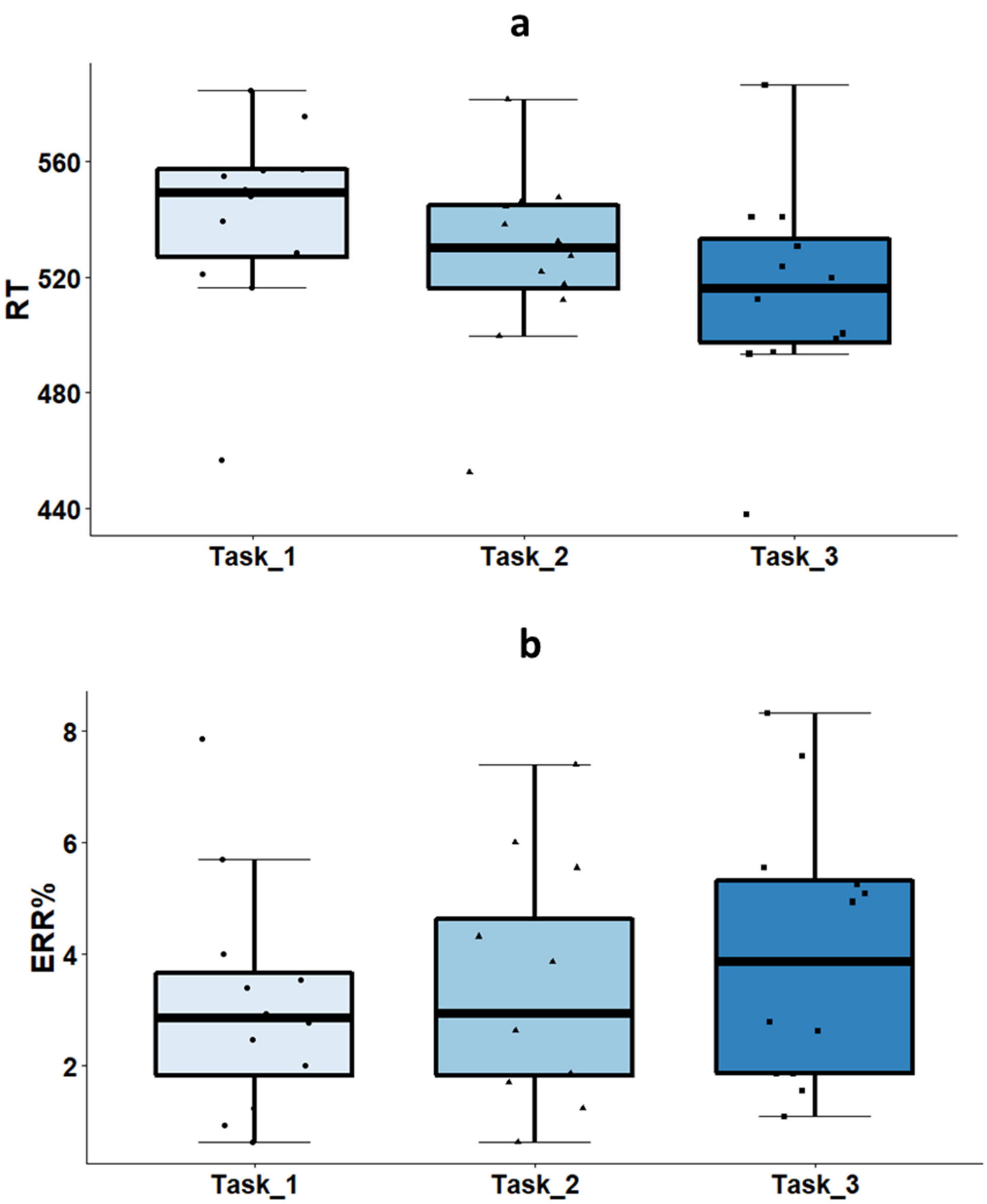

Figure S1. Subjects' Performances (n=12) assessed during the three blocks of the Simon Task. Boxplots show Reaction Times (RT) in the upper panel (a) and Error Rates (ERR%) in the lower panel (b).
